# Assessing Quality of Life and Patient-Reported Outcomes in Diabetic Macular Edema Treated With Anti-vascular Endothelial Growth Factor (Anti-VEGF) Agents: A Systematic Review and Meta-Analysis

**DOI:** 10.7759/cureus.95469

**Published:** 2025-10-26

**Authors:** Fathimath J Shameem, Francis Asante Baadu, Sridevi Venugopal, Chinenye Iguh, Marwah M Hafiz, Maheen Zahid, Muhammad Azhar Uddin

**Affiliations:** 1 Plastic Surgery, Aberdeen Royal Infirmary, Aberdeen, GBR; 2 Internal Medicine, Komfo Anokye Teaching Hospital, Kumasi, GHA; 3 Ophthalmology, Government Cuddalore Medical College and Hospital, Annamalai Nagar, IND; 4 Medicine, Windsor University School of Medicine, Cayon, KNA; 5 Ophthalmology, Pakistan Institute of Medical Sciences, Islamabad, PAK; 6 Ophthalmology, King Edward Medical University, Lahore, PAK; 7 General Surgery, The Queen Elizabeth Hospital King’s Lynn, King’s Lynn, GBR

**Keywords:** anti-vegf therapy, diabetic macular edema (dme), national eye institute visual function questionnaire - 25, nei vfq-25, patient-reported outcomes measures, quality of life (qol)

## Abstract

Diabetic macular edema (DME) is a leading cause of vision impairment, with profound effects on patients’ quality of life (QoL). Anti-vascular endothelial growth factor (anti-VEGF) agents are the current standard of care, but their impact on patient-reported outcomes (PROMs) remains incompletely understood. We conducted a systematic review and meta-analysis of five randomized controlled trials (RCTs) and one cohort study involving 1,793 participants, evaluating the effects of anti-VEGF therapy compared with sham injections or laser therapy on QoL as measured by the National Eye Institute Visual Function Questionnaire (NEI VFQ-25). Pooled analysis demonstrated a statistically significant improvement in composite QoL scores with anti-VEGF therapy (mean difference 3.11; 95% CI 1.89-4.33; p<0.00001), although the absolute magnitude of improvement was modest. Subgroup analysis revealed greater benefit compared with sham injections than with laser therapy, and long-term follow-up (24 months) showed more pronounced gains compared with shorter durations. Sensitivity analysis excluding the non-randomized study confirmed the robustness of these findings. While anti-VEGF therapy improves QoL in patients with DME, real-world barriers such as treatment burden, access, and cost may limit patient-perceived benefits. Future research should integrate patient-centered approaches to better capture the treatment impact beyond visual outcomes.

## Introduction and background

Diabetic macular edema (DME) represents one of the most common and vision-threatening complications of diabetic retinopathy, and is currently a leading cause of visual impairment among working-age adults worldwide. Recent estimates suggest that approximately 21 million people are affected globally, reflecting the escalating prevalence of diabetes and its associated microvascular complications [1]. Beyond the direct impact on vision, DME imposes a substantial burden on patients’ independence, productivity, and psychosocial well-being, ultimately contributing to diminished quality of life (QoL) and increased socioeconomic costs [2,3].

For more than two decades, focal and grid laser photocoagulation (established by the Early Treatment Diabetic Retinopathy Study) was regarded as the standard of care [4]. However, the procedure primarily reduced the risk of further visual loss without restoring vision. The therapeutic paradigm shifted with the introduction of intravitreal anti-vascular endothelial growth factor (anti-VEGF) agents, including ranibizumab, aflibercept, and off-label bevacizumab. Pivotal randomized controlled trials (RCTs), such as Ranibizumab Monotherapy or Combined with Laser versus Laser Monotherapy for Diabetic Macular Edema (RESTORE), Diabetic Retinopathy Clinical Research Network (DRCR.net), Intravitreal Ranibizumab or Triamcinolone in Combination with Laser Photocoagulation for Diabetic Macular Edema (Protocol I), Comparative Effectiveness Trial of Aflibercept, Bevacizumab, and Ranibizumab for DME Treatment (Protocol T), Intravitreal Aflibercept Injection in Vision Impairment due to DME (VIVID-DME), and Study of Intravitreal Aflibercept Injection in Patients with Diabetic Macular Edema (VISTA-DME), consistently demonstrated that anti-VEGF therapy provides superior improvements in best-corrected visual acuity (BCVA) and central retinal thickness compared with laser or sham treatment [5-7]. Consequently, anti-VEGF injections are now recommended as first-line therapy for center-involving DME in major clinical guidelines.

While BCVA and anatomic outcomes remain the primary efficacy endpoints in clinical trials, these measures do not fully capture the lived experiences of patients. Patient-reported outcome measures (PROMs) have therefore gained increasing importance in ophthalmology, as they reflect broader domains such as functional vision, emotional well-being, and social participation [8]. Among these, the National Eye Institute Visual Function Questionnaire (NEI VFQ-25) is the most widely used instrument in DME trials, with well-documented validity and responsiveness [9]. Importantly, systematic reviews suggest that even modest improvements in patient-reported outcome (PRO) scores may correspond to meaningful clinical and psychosocial benefits [10].

Nevertheless, the impact of anti-VEGF therapy on QoL remains inconclusive. For instance, RESTORE and DRCR.net Protocol I reported significant improvements in vision-related QoL in parallel with visual gains [11,12], whereas trials such as Ranibizumab 0.5 mg for Diabetic Macular Edema with Bimonthly Monitoring after a Phase of Initial Treatment (RELIGHT) found inconsistent or domain-specific benefits [13]. Furthermore, real-world evidence highlights that treatment outcomes are influenced by factors extending beyond efficacy, including the burden of frequent injections, financial constraints [14], and challenges with long-term adherence [15], particularly in resource-limited settings [16]. These considerations underscore the need for a comprehensive synthesis of available evidence to clarify the broader value of anti-VEGF therapy from the patient perspective.

Accordingly, this systematic review and meta-analysis aims to evaluate the impact of intravitreal anti-VEGF therapy, compared with non-anti-VEGF interventions, on QoL and PROs in individuals with DME. By integrating data from both RCTs and real-world studies, this review seeks to inform clinical decision-making and health policy regarding the patient-centered benefits of anti-VEGF treatment beyond traditional visual acuity outcomes.

## Review

Materials and methods

A comprehensive literature search was conducted in PubMed, the Cochrane Library, and Google Scholar to identify studies assessing PROMs and QoL in adults with DME treated with intravitreal anti-VEGF agents. No date restrictions were applied during the search, and the last search was conducted on July 20, 2025. Search terms included combinations of “diabetic macular edema,” “anti-VEGF,” “ranibizumab,” “aflibercept,” “bevacizumab,” “faricimab,” “brolucizumab,” “quality of life,” “NEI VFQ-25,” “patient-reported outcomes,” “PROM,” “EQ-5D,” and “SF-36.” Reference lists of included articles and relevant reviews were manually screened to identify additional eligible studies. Grey literature, including unpublished studies, trial registries, and conference abstracts, was not systematically searched due to resource constraints. The review focused on peer-reviewed, indexed publications to maintain methodological rigor.

Eligibility criteria

Eligible studies included English-language publications enrolling adults (≥18 years) with DME treated with intravitreal anti-VEGF agents (ranibizumab, aflibercept, bevacizumab, faricimab, or brolucizumab) and reporting at least one validated PROM (e.g., NEI VFQ-25, EuroQol-5 Dimensions (EQ-5D), Short Form 36-item Health Survey (SF-36)) or QoL measure. RCTs, observational designs, and real-world studies were included. Studies including mixed retinal disease populations were only eligible if subgroup data specific to DME were reported. In such cases, we extracted and analyzed the DME-specific results separately. Studies were excluded if they lacked PRO/QoL data, focused solely on anatomical or visual outcomes, investigated non-DME retinal diseases without DME-specific results, or were animal studies, case reports, editorials, commentaries, or non-English publications (Table 1).

**Table 1:** Inclusion and exclusion criteria VEGF: Anti-vascular Endothelial Growth Factor; PRO: Patient reported outcome; QoL: Quality of life; NEI VFQ-25: National Eye Institute Visual Function Questionnaire; EQ-5D: EuroQol-5 Dimensions; SF-36: Short Form 36-item Health Survey; RCT: Randomized controlled trial.

Category	Inclusion criteria	Exclusion criteria
Population	Adults (≥18 years) with a diagnosis of DME	Patients <18 years; studies focusing solely on other retinal diseases without separate DME-specific results
Intervention	Treated with intravitreal anti-VEGF agents: ranibizumab, aflibercept, bevacizumab, faricimab, or brolucizumab	Studies not involving anti-VEGF treatment
Outcomes	Reported at least one validated PRO or QoL measure (e.g., NEI VFQ-25, EQ-5D, SF-36)	Studies lacking PRO/QoL data or reporting only anatomical/visual outcomes
Study design	RCTs, observational studies, or real-world studies	Case reports, case series, animal studies, reviews, editorials, commentaries, conference abstracts
Language & publication type	English-language, peer-reviewed full-text articles	Non-English publications

Study Selection

Two reviewers (C.I. and M.M.H) independently screened titles and abstracts to identify potentially eligible studies. Full-text articles were retrieved for all studies meeting inclusion criteria or when eligibility was uncertain. Discrepancies regarding study eligibility or data extraction were resolved through discussion or by a third reviewer (M.Z.). The selection process followed the Preferred Reporting Items for Systematic Reviews and Meta-Analyses (PRISMA) guidelines [17], and a PRISMA flow diagram was constructed to illustrate the number of studies identified, screened, excluded, and included.

Data Extraction

A standardized data extraction form was used to collect study-level information, including first author, year of publication, country or setting, study design, sample size, mean age, sex distribution, intervention and comparator details, follow-up duration, QoL/PRO instrument used, and reported outcome values (mean ± standard deviation (SD), standard error (SE), or confidence interval (CI)). For studies with multiple follow-up time points, the primary endpoint was defined according to the authors’ specification (most commonly six or 12 months).

Risk of Bias Assessment

The Cochrane Risk of Bias Tool was applied to RCTs, evaluating five domains: random sequence generation, allocation concealment, blinding of participants and personnel, completeness of outcome data, and selective reporting. Two reviewers (M.A.U. and C.I.) performed the assessment independently, with disagreements resolved by consensus. Risk of bias results were summarized narratively and graphically. For the included observational study, risk of bias was assessed using the Newcastle-Ottawa Scale (NOS) [18], which evaluates selection, comparability, and outcome domains.

Data Synthesis and Statistical Analysis

Where at least two studies reported the same outcome measure, a meta-analysis was performed using mean differences (MD) or standardized mean differences (SMD) with corresponding 95% CIs. Heterogeneity was assessed using the I² statistic and chi-square test. A random-effects model was applied when heterogeneity exceeded 50%; otherwise, a fixed-effect model was used. Subgroup analyses were planned based on the type of anti-VEGF agent, follow-up duration, and baseline visual acuity. All analyses were conducted using RevMan 5.4 (The Cochrane Collaboration, London, England, UK).

Results

**Figure 1:**
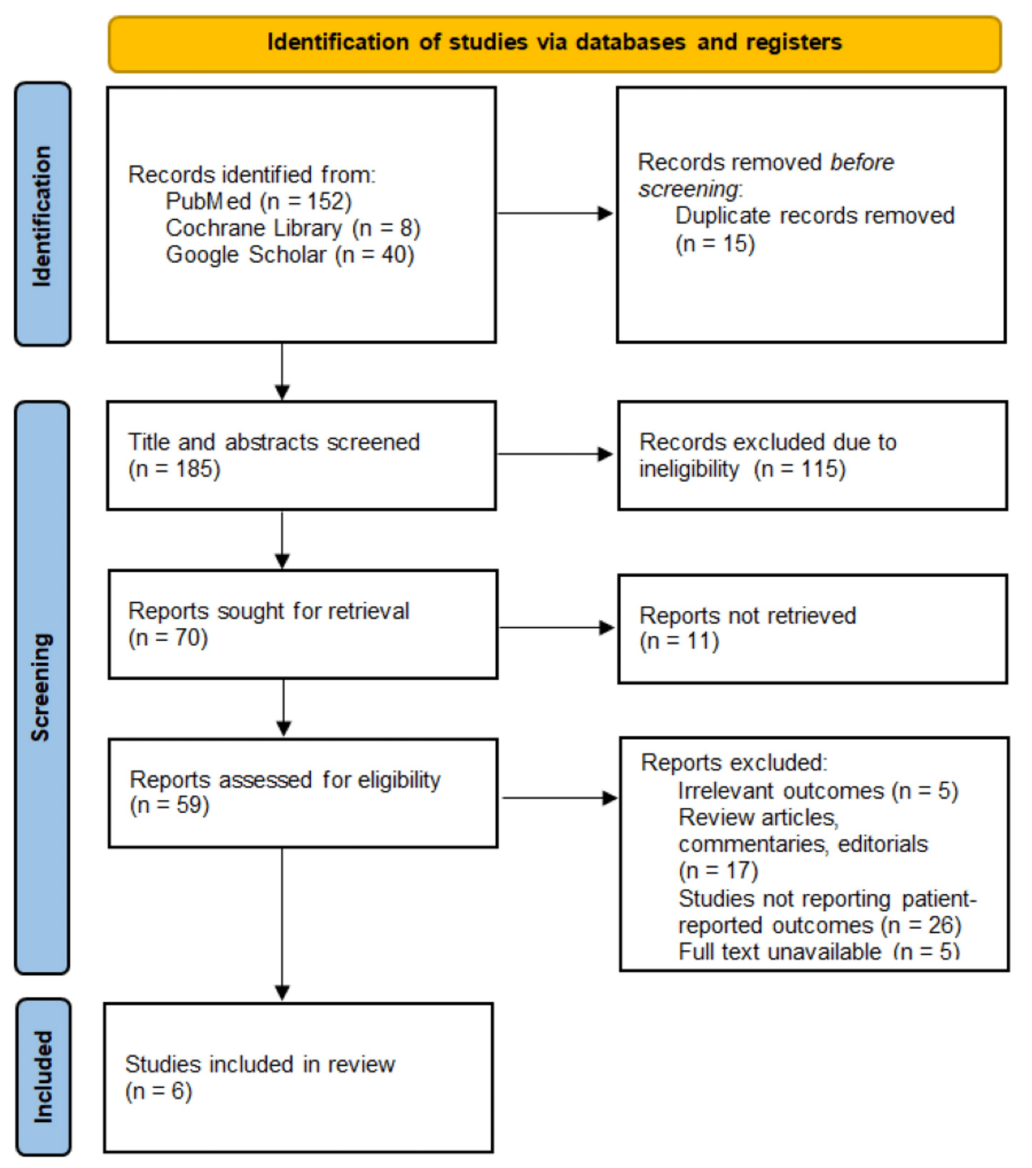
PRISMA flow diagram 2020 PRISMA: Preferred Reporting Items for Systematic Reviews and Meta-Analyses [17].

A total of 200 records were retrieved from database searches, including PubMed (n=152), Cochrane Library (n=8), and Google Scholar (n=40). After removing 15 duplicates, 185 records were screened by title and abstract, resulting in 115 exclusions for irrelevance. Seventy articles were sought for full-text retrieval, of which 11 could not be obtained. Of the 59 articles assessed for eligibility, five were excluded for irrelevant outcomes, 17 were review or commentary articles, 26 did not report PROs, and five did not have full texts available. Ultimately, six studies met the inclusion criteria for the meta-analysis (Figure 1).

Study Characteristics

The six included studies, published between 2011 and 2023, were predominantly multicenter RCTs. Sample sizes ranged from 70 to 759 participants. All studies investigated intravitreal ranibizumab (0.5 mg) as the anti-VEGF intervention, compared against either sham injections or laser photocoagulation. Follow-up durations varied from six to 24 months. Mean participant ages were in the early to mid-60s across studies, and the proportion of male participants ranged from 51% to 63%. All trials used the NEI VFQ-25 or its cultural adaptation to assess QoL and vision-related PROs (Table 2).

**Table 2:** Summary of the key characteristics of eligible studies included in the review Anti-VEGF: Anti-vascular Endothelial Growth Factor; PRO: Patient reported outcome; QoL: Quality of life; NEI VFQ-25: National Eye Institute Visual Function Questionnaire; EQ-5D: EuroQol-5 Dimensions; SF-36: Short Form 36-item Health Survey; RCT: Randomized controlled trial; OCEAN: Observation of treatment patterns with LuCEntis and real-life ophthalmic monitoring, including optional OCT in Approved iNdications.

Author	Setting	Study design	Sample size	Mean age ± SD (Year)	Anti-VEGF agent(s)	Comparator	Duration of follow-up	QoL/PRO tool used
Mitchell et al., 2011 [5]	Multicenter	RCT	Intervention group: 116; Control group: 111	Intervention group: 62.9 ± 9.29; Control group: 63.5 ± 8.81	Ranbizumab (0.5 mg)	Laser photocoagulation	12 months	NEI VFQ-25
Bressler et al., 2014 [19]	USA	RCT	Intervention group: 251; Control group: 253	63.6 ± 9.7	Ranibizumab (0.5 mg)	Sham injections	24 Months	NEI VFQ-25
Mitchell et al., 2015 [20]	Multicenter	RCT	Intervention group: 83; Control group: 74	62.6 ± 8.8	Ranbizumab (0.5 mg)	Laser monotherapy	12 months	NEI VFQ-25
Turkoglu et al., 2015 [21]	Turkey	RCT	Intervention group: 35; Control group: 35	Intervention group: 64.6 ± 8.2 Control group: 63.8 ± 7.4	Ranibizumab (0.5 mg)	Focal/grid laser	6 months	VFQ-25
Suner et al., 2017 [22]	USA	RCT	Intervention group: 252; Control group: 257	62.7 ± 10.7	Ranibizumab (0.5 mg)	Sham injections	24 Months	NEI VFQ‑25
Schuster et al., 2023 [23]	Germany	Cohort	Intervention group: 176; Control group: 150	74.5 ± 10.9	Ranibizumab, Aflibercept	Historical OCEAN data	12 months	NEI VFQ-25

**Figure 2:**
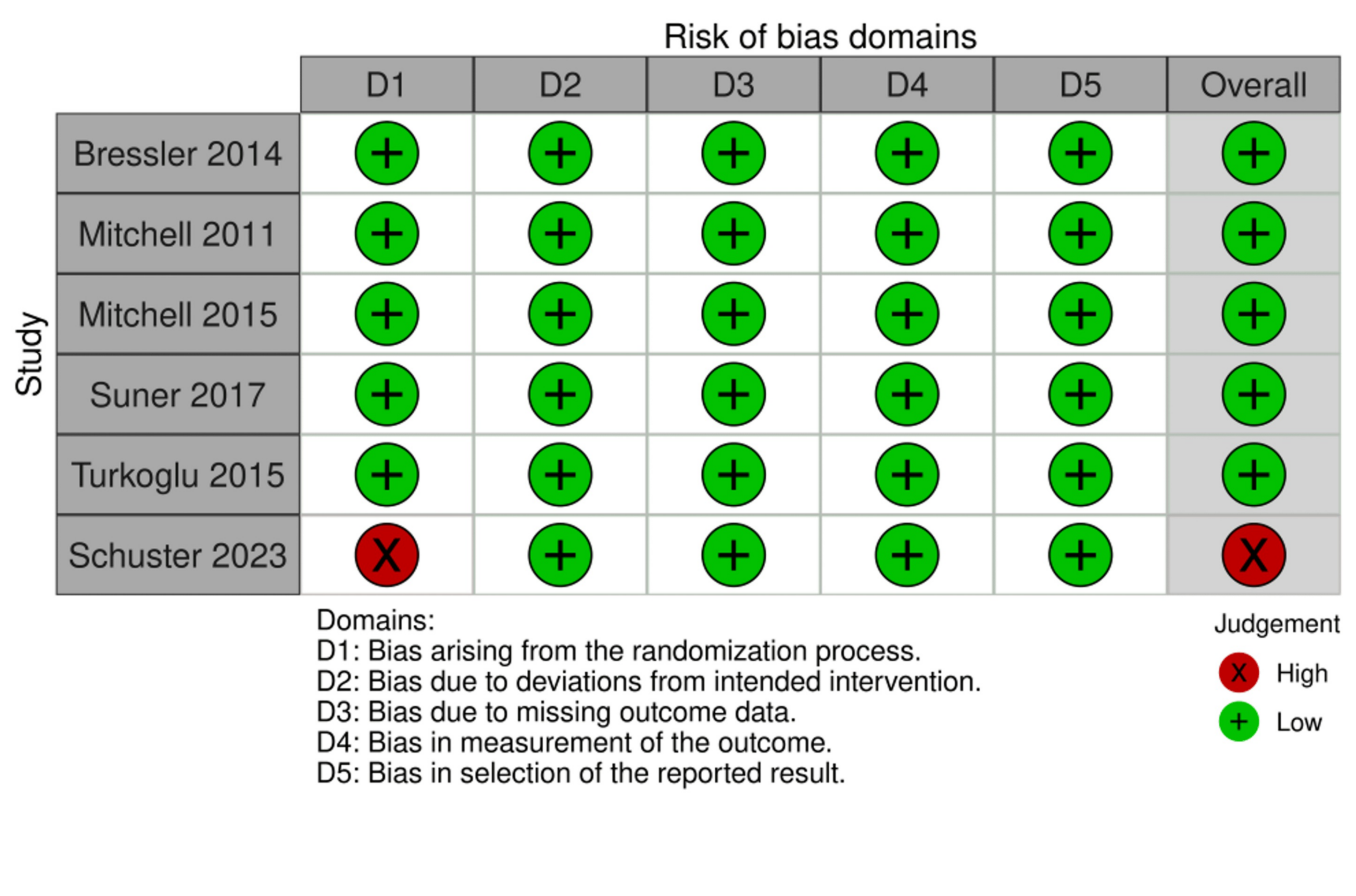
Risk of bias assessment [5,19-23]

Among the five RCTs, four demonstrated low risk of bias across all five domains and were therefore judged to have an overall low risk of bias. In contrast, one was rated as having a high risk of bias in Domain 1 (bias arising from the randomization process), while all other domains were judged to be at low risk. Consequently, this study was classified as having an overall high risk of bias (Figure 2).

The included cohort study was assessed using the NOS and received a total score of seven out of eight, indicating moderate-to-high methodological quality.

Primary Analysis

**Figure 3:**
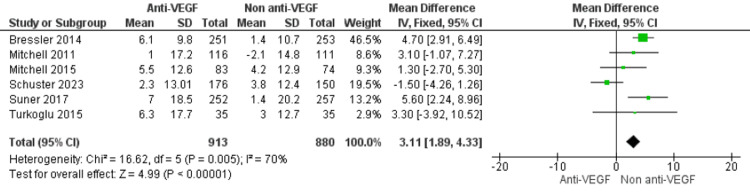
Forest plot of the primary analysis demonstrating the effect of anti-VEGF therapy on QoL measures compared with non–anti-VEGF interventions in DME Anti-VEGF: Anti-vascular Endothelial Growth Factor; QoL: Quality of life; DME: Diabetic macular edema. [5,19-23]

This meta-analysis included five RCTs and one cohort study, encompassing 1,793 participants (913 receiving anti-VEGF agents and 880 receiving non-anti-VEGF treatments). Overall, anti-VEGF therapy led to a statistically significant improvement in QoL scores compared with non-anti-VEGF interventions (MD=3.11; 95% CI: 1.89 to 4.33; p<0.00001). However, there was substantial heterogeneity among studies (I²=70%) (Figure 3).

Subgroup Analysis by Comparator

**Figure 4:**
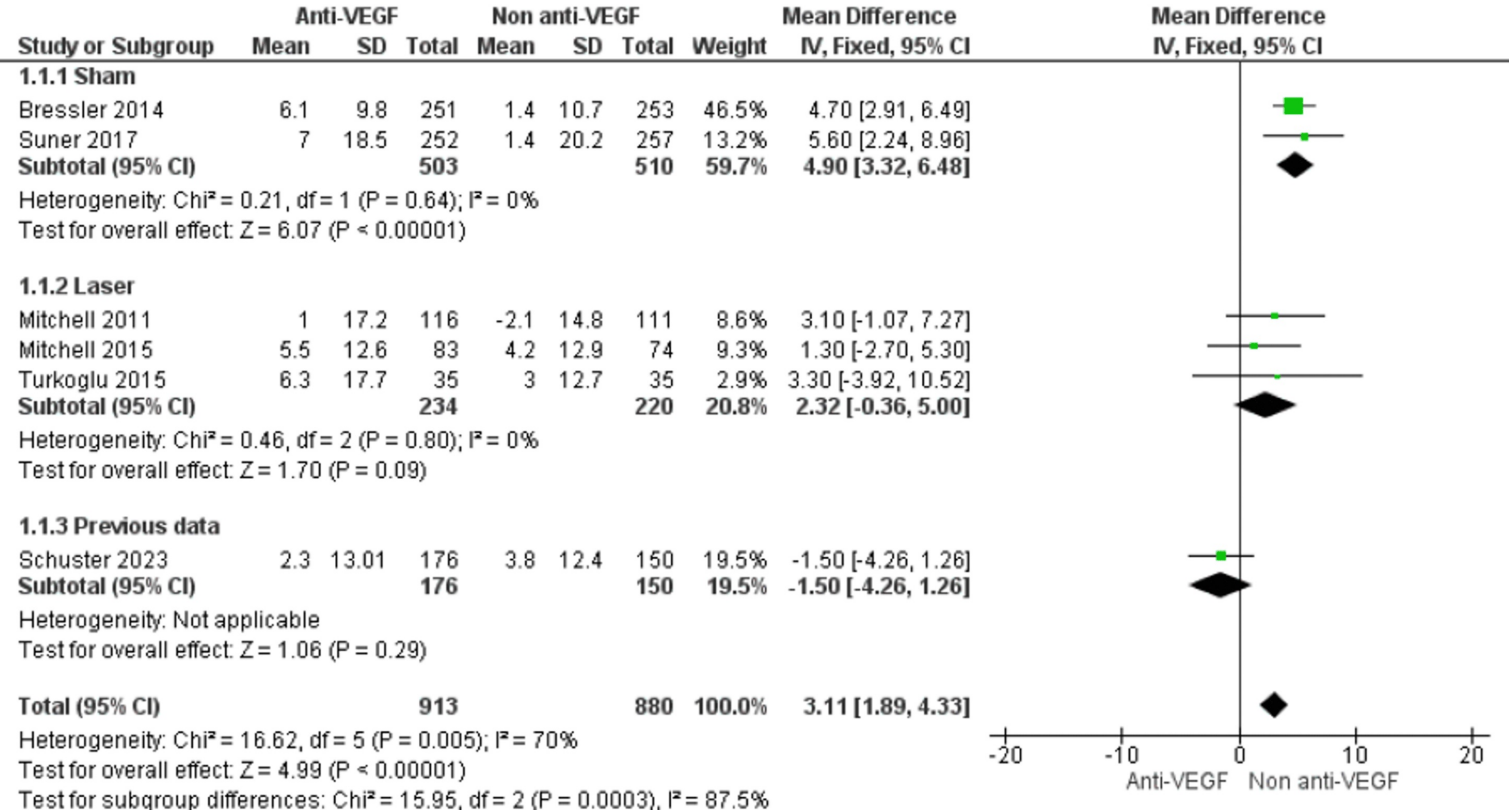
Forest plot of subgroup analyses according to comparator type, showing the effect of anti-VEGF therapy versus non–anti-VEGF interventions on patient-reported QoL outcomes in DME Anti-VEGF: Anti-vascular Endothelial Growth Factor; QoL: Quality of life; DME: Diabetic macular edema. [5,19-23]

When examined by comparator type, the benefit of anti-VEGF therapy was most pronounced against sham injections, showing an average improvement of nearly five points on the QoL scale (MD=4.90; 95% CI: 3.32 to 6.48; p<0.00001; I²=0%). Compared with laser therapy, the effect was smaller and not statistically significant (MD=2.32; 95% CI: -0.36 to 5.00; p=0.09; I²=0%). The cohort study using historical controls [21] showed no meaningful difference between groups (MD=-1.50; 95% CI: -4.26 to 1.26; p=0.29). Differences between subgroups were statistically significant (χ²=15.95, p=0.0003; I²=87.5%) (Figure 4).

Subgroup Analysis by Duration of Follow-up

**Figure 5:**
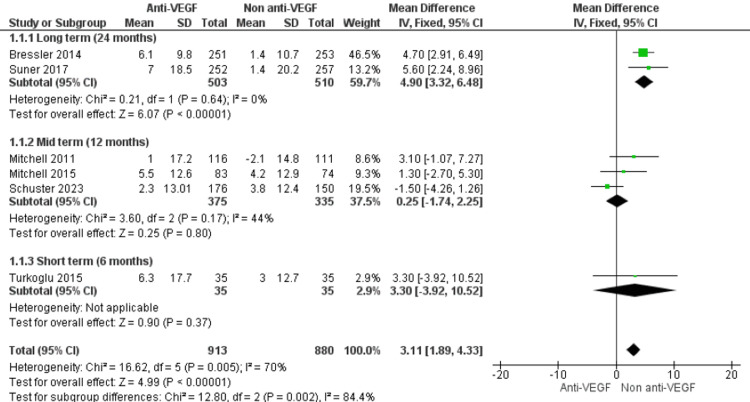
Forest plot of subgroup analyses according to follow-up duration, comparing the effect of anti-VEGF therapy versus non–anti-VEGF interventions on patient-reported QoL outcomes in DME Anti-VEGF: Anti-vascular Endothelial Growth Factor; QoL: Quality of life; DME: Diabetic macular edema. [5,19-23]

A subgroup analysis was performed to examine whether the duration of follow-up influenced the impact of anti-VEGF therapy on quality of life in patients with diabetic macular edema. Studies were categorized as short-term (six months), mid-term (12 months), or long-term (24 months). In the long-term subgroup (two studies, n=510), anti-VEGF therapy was associated with a statistically significant improvement in QoL scores compared with non-anti-VEGF treatment (MD=4.90, 95% CI 3.26 to 6.48; p<0.00001), with no heterogeneity (I²=0%). In the mid-term subgroup (three studies, n=351), there was no statistically significant difference between anti-VEGF and control (MD=0.25, 95% CI -1.74 to 2.25; p=0.80), with moderate heterogeneity (I²=44%). In the short-term subgroup (one study, n=35), the pooled effect was also non-significant (MD=3.30, 95% CI -3.92 to 10.52; p=0.37). The test for subgroup differences indicated a statistically significant variation in treatment effect across follow-up durations (χ²=12.80, df=2, p=0.002), with the greatest benefit observed in the long-term subgroup (Figure 5).

Sensitivity Analyses

**Figure 6:**
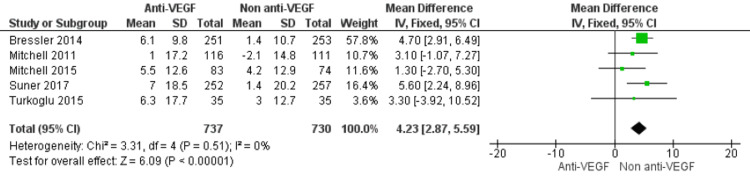
Forest plot of sensitivity analyses assessing the robustness of pooled estimates for anti-VEGF therapy versus non–anti-VEGF interventions on QoL outcomes in DME Anti-VEGF: Anti-vascular Endothelial Growth Factor; QoL: Quality of life; DME: Diabetic macular edema. [5,19-23]

Sensitivity analysis, excluding the non-randomized cohort study, confirmed the robustness of the findings. In the pooled analysis of five RCTs (n=1,467), anti-VEGF treatment continued to demonstrate a marked and statistically significant benefit over non-anti-VEGF therapy (MD=4.23; 95% CI: 2.87 to 5.59; p<0.00001), with no evidence of heterogeneity (I²=0%) (Figure 6).

Publication Bias

**Figure 7:**
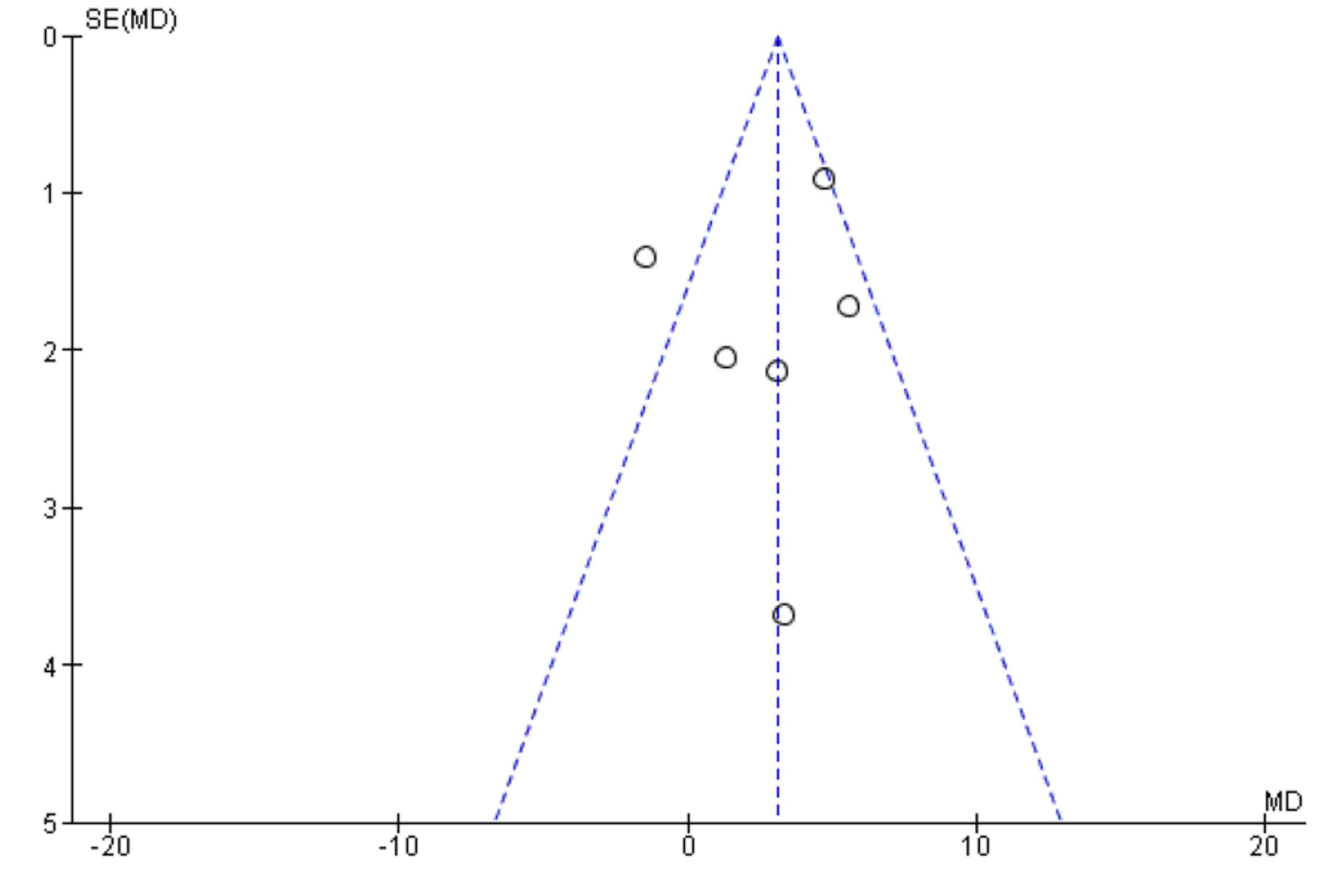
Funnel plot assessing publication bias among studies evaluating anti-VEGF therapy versus non–anti-VEGF interventions on QoL outcomes in DME Anti-VEGF: Anti-vascular Endothelial Growth Factor; QoL: Quality of life; DME: Diabetic macular edema.

Visual inspection of the funnel plot suggested approximate symmetry, with no clear indication of small-study effects. Given the small number of studies (k=6), statistical tests for funnel plot asymmetry (Egger’s or Begg’s) were not performed, as they are underpowered and unreliable in this context (Figure 7).

Discussion

This meta-analysis provides evidence that anti-VEGF therapy, particularly ranibizumab, is associated with meaningful improvements in PRO in individuals with DME. Across five RCTs, patients receiving anti-VEGF agents reported significantly better vision-related function compared with those receiving non-anti-VEGF treatments, with the greatest gains observed in comparison with sham injections and in long-term follow-up studies.

Our findings align with previous clinical trials and observational studies demonstrating the value of anti-VEGF therapy in improving not only visual acuity but also PROs. The Vision-Related Quality of Life in Patients with Diabetic Macular Edema Treated with Intravitreal Aflibercept (AQUA) study reported that aflibercept treatment led to sustained improvements in NEI VFQ-25 scores over one year of follow-up, confirming the impact of anti-VEGF agents on patient-centered measures of visual function [24]. Similarly, Granström et al. [25] demonstrated that real-world treatment with anti-VEGF correlated with significant gains in both NEI VFQ-25 and generic QoL instruments such as the SF-36, reinforcing the clinical meaningfulness of our pooled findings.

The pronounced benefit seen in sham-controlled studies is consistent with evidence that anti-VEGF agents offer substantial advantages over observation alone. However, the lack of a significant difference compared with laser therapy reflects the more modest incremental benefits of ranibizumab when compared with established, albeit less effective, treatments. It has been demonstrated that ranibizumab with deferred laser improved visual outcomes more than laser alone, but the QoL benefit was smaller in magnitude at early time points [26]. This suggests that while laser therapy still confers some functional benefit, anti-VEGF remains superior for longer-term outcomes.

An important observation from our subgroup analysis was that treatment effect differed by follow-up duration, with the strongest benefit observed at 24 months, but limited or no benefit at six to 12 months. This time-dependent effect underscores the importance of sustained treatment exposure to achieve meaningful functional gains. Similar findings were reported by Mitchell et al. [5] in the RESTORE trial, where ranibizumab-treated patients reported greater QoL improvements over two years compared with controls. Long-term real-world evidence also supports this trend. Wen et al. [27] found that adherence to regular anti-VEGF therapy was crucial in maintaining both visual acuity and QoL benefits, whereas irregular treatment schedules often diminished patient-perceived gains. Conversely, some mid-term studies have reported a weaker association between short-term visual acuity gains and QoL [13], highlighting that QoL improvements may lag behind visual improvements or require sustained stabilization to be appreciated by patients.

Our sensitivity analysis confirmed that exclusion of the non-randomized study by Schuster et al. [23] did not materially alter the results. The absence of a QoL benefit in that cohort likely reflects methodological limitations, including reliance on historical controls and potential residual confounding. This highlights the importance of rigorous RCT-based evidence in evaluating QoL in DME.

Although statistically significant, the absolute magnitude of QoL improvement in NEI VFQ-25 scores was modest. Nonetheless, prior reviews emphasize that even small improvements in QoL may represent clinically meaningful changes for patients [28,29]. To contextualize the observed improvement, prior studies have estimated that a change of approximately three to five points on the NEI VFQ-25 composite score represents the minimal clinically important difference (MCID) for patients [30]. In our meta-analysis, the pooled mean difference was 3.11 points (95% CI 1.89-4.33), which lies at the lower end of this threshold. This suggests that anti-VEGF therapy provides not only a statistically significant but also a potentially clinically meaningful improvement in QoL, particularly when considered alongside the durability of benefit observed at longer follow-up durations. Moreover, qualitative evidence suggests that treatment burden, including frequent injections and clinic visits, can counterbalance perceived benefits [6]. Real-world studies in resource-limited settings have also highlighted systemic barriers to regular treatment, including cost and accessibility, which directly influence patient-reported satisfaction [31]. Taken together, these factors suggest that QoL gains may depend as much on treatment logistics and patient experience as on clinical efficacy.

This meta-analysis is the first synthesis focusing specifically on PROs in DME treated with anti-VEGF therapy, addressing an important gap in the literature where clinical and anatomical endpoints have traditionally dominated. The included studies were largely multicenter RCTs with low risk of bias, and all employed validated QoL instruments (NEI VFQ-25 or its cultural adaptations), ensuring methodological rigor and comparability. In addition, sensitivity analyses confirmed the robustness of findings when the non-randomized evidence was excluded.

However, this review has several limitations. The number of included studies was small, limiting the precision of subgroup analyses and precluding formal assessment of publication bias. Considerable heterogeneity in the primary analysis may reflect differences in populations, comparators, and follow-up durations. While NEI VFQ-25 is a validated measure of vision-related function, it may not capture broader aspects of health-related QoL. Treatment burden and adherence, important determinants of real-world patient experience, were variably reported. Additionally, the review was limited to English-language studies indexed in PubMed, Cochrane Library, and Google Scholar, and grey literature was not included, which may have introduced language and publication bias and omitted potentially relevant studies.

## Conclusions

This meta-analysis suggests that intravitreal anti-VEGF therapy leads to significant, albeit modest, improvements in QoL and PROMs among individuals with DME. The greatest benefits were observed in sham-controlled and long-term studies (24 months), underscoring the importance of sustained treatment exposure to achieve meaningful functional gains. Although sensitivity analyses support the robustness of our findings, the modest absolute improvements in QoL, combined with real-world factors such as treatment burden, limited access, and cost, suggest that the overall patient-perceived benefit of anti-VEGF therapy extends beyond changes in clinical outcomes alone. Future research should prioritize patient-centered approaches, integrating qualitative measures and long-term follow-up, to optimize both outcomes and treatment experience in this growing patient population.
